# Therapeutic potential of *Mucuna pruriens *(Linn.) on high-fat diet-induced testicular and sperm damage in rats 

**DOI:** 10.22038/AJP.2022.20261

**Published:** 2022

**Authors:** Anuradha Murugesan, Karthik Ganesh Mohanraj, Khayinmi Wungpam Shimray, Mohammad Zafar Iqbal Khan, Prakash Seppan

**Affiliations:** 1 *Department of Anatomy, Dr. Arcot Lakshmanaswamy Mudaliar Postgraduate Institute of Basic Medical Sciences, University of Madras, Taramani Campus, Chennai, India*

**Keywords:** Hypercholesterolemia, Mucuna pruriens, Testis, Spermatogenesis, Oxidative stress

## Abstract

**Objective:**

*Mucuna pruriens* Linn., a leguminous plant, is identified as a herbal medicine for improving fertility-related disorders in the alternative and complementary systems of medicine. The study was focused on evaluating the therapeutic potential of *M. pruriens* on testis and sperm parameters in a high-fat-induced hypercholesterolemia model.

**Materials and Methods:**

Male rats were divided as normal-control rats (NCR); normal-control rats + *M.pruriens* (200 mg/kg b.w. of ethanolic extract of *M. pruriens* seed) treated (NCRD); hypercholesterolemic rats (HCR) and hypercholesterolemic rats + *M. pruriens* (HCRD). Groups were further divided into three post-exposure periods (subgroups) of 9, 18, and 36 days, and the progressive changes in testis histology and sperm were analyzed.

**Results:**

The study showed a significant impairment in testicular histoarchitecture, depletion of antioxidant enzyme levels, increased oxidative stress and lipid peroxidation in the HCR group. The study indicated severe structural and functional damage in sperm parameters and diminished chromatin integrity in the HCR group. In the HCR rats, the follicular stimulating hormone (FSH) and luteinizing hormone (LH) and testosterone were significantly reduced. There was a significant improvement in sperm parameters and testis histology in the HCRD group.

**Conclusion:**

The study reveals the potential efficacy of *M. pruriens* to improve spermatogenesis, sperm parameters and hormone levels in hypercholesterolemic rats.

## Introduction

*Mucuna pruriens* Linn., a leguminous plant, is used in Indian traditional medicine to improve male fertility (Sathiyanarayanan et al., 2007 ▶). This plant was used extensively in traditional Ayurveda, which was practiced since the Vedic period, i.e. 1500-1000 BC, to treat male infertility and neurological disorders (Lampariello et al., 2012 ▶); however, without proper scientific validation. *M. pruriens* is found in more than 200 indigenous drug formulations and all parts of the plant show medicinal properties (Pandey, 1998 ▶; Pandey, 1999 ▶). *M. pruriens* is rich in alkaloids, including prurieninine, prurienine and prurienidine (Misra et al., 2004 ▶). The seeds contain levodopa (L-DOPA) (Siddhuraju et al., 1996 ▶) and amino acids like methionine, tyrosine, lysine, glycine, aspartic acid, glutamic acid, leucine and serine along with globulins and albumins (Pant and Joshi, 1970). Besides, fatty acids, carbohydrates, and related compounds such as oleic acid, linoleic acid and palmitic acid were isolated from the plant (Adebowale et al., 2005 ▶). 

*M. pruriens* has been recognized as an aphrodisiac agent (Suresh and Prakash, 2012 ▶). *M. pruriens* seed ethanolic extract was able to protect or recover sperm structural stability from oxidative stress, and improved fertility in aged and diabetic rats (Suresh et al., 2013 ▶; Suresh et al., 2010 ▶). Further, the extract has shown androgenic influence in treating aging-induced reproductive disorders (Seppan et al., 2011 ▶). The aphrodisiac and spermatogenic activities have been attributed to L-DOPA presence (Pulikkalpura et al., 2015 ▶). The seed of *M. pruriens* has shown anti-lipid peroxidation property, mediated through the removal of superoxides and hydroxyl radicals (Misra and Wagner, 2005 ▶). The anti-cholesterolemia effect of extract of *M. pruriens* seed could be due to its high flavonoid and high antioxidant activity that exert protection on circulating and membrane lipids by preserving endogenous antioxidants (Ratnawati and Widowati, 2011 ▶). However, the efficacies of *M. pruriens* seed in lowering the lipids profile are not known. 

Hypercholesterolemia is an asymptomatic disorder which refers to abnormally high levels of lipids in the serum plasma, mainly cholesterol and triglycerides (Skeldon et al., 2015 ▶), caused prominently by dietary intake individual's physical activities, environmental conditions, and stress, thereby a common lifestyle disorder (Kannan et al., 2019 ▶). Consuming a high-fat diet during the juvenile/adolescent period harms numerous bodily structures and functions (Hurt et al., 2010 ▶). The impacts of high-fat diets or obesity on the male reproductive system can induce testicular and epididymal dysfunction (Sedes et al., 2018 ▶) and could set to infertility in adults (Saez and Drevet, 2019 ▶). 

The known activities through the rich source of bioactive molecules in the ethanolic extract of the *M. pruriens* seed have been motivating aspects to critically analyze testicular dysfunction, sperm DNA integrity, and mitochondrial membrane potential under one of the dreadful pathophysiological conditions such as hypercholesterolemia, which was not reported. 

## Materials and Methods

**Animals used and experimental design**

Seventy-two adult healthy male Wistar albino rats aged around four months (body weight: 250 to 300 g) were used for the study. Animals were randomly divided into the following groups with eighteen animals per group; Normal-Control rats (NCR), Normal-Control rats + *M.pruriens* treated (NCRD); Hypercholesterolemic rats (HCR), and Hypercholesterolemic rats + *M. pruriens *treated (HCRD). Randomly selected six animals from each of the above-mentioned groups were sacrificed on the 9^th^, 18^th^, and 36^th^ days (sub-groups) from induction day. The data were collected from these sub-groups to delineate the changes during the high-fat diet exposure.

The animals were procured from the central animal house facility, University of Madras. The rats were housed individually in a polypropylene cage, and maintained under a controlled environment at room temperature (23±2°C), humidity (50±5%), and a 12:12 light/dark cycle, under standard laboratory conditions with water *ad libitum* during the study. The animal study protocol was approved by the institutional animal ethical committee, Dr.A.L.M. PGIBMS, University of Madras (IAEC No. 03/02/2011). Animal maintenance, experiments and euthanasia were according to the guidelines of the Canadian Council for Experimental Animal Care (Olfert et al., 1993 ▶) and The Committee for the Purpose of Control and Supervision of Experiments on Animals (CPCSEA), India, guidelines for laboratory animal facility (2003).

**Animal model creation**

The model creation was done by feeding a high-fat diet that includes standard rodent chow powder (62.75%), cholic acid (0.25%), cholesterol (2%), lard oil (15%), wheat flour (10%), and sucrose (10%) for six weeks to induce hypercholesterolemia (Garjani et al., 2011 ▶).

**Drug preparations**

The ethanolic extract of *M. pruriens* seed was used in the present study. Details of the extract preparation are described elsewhere (Suresh et al., 2009 ▶; Prakash et al., 2018 ▶). Briefly, the seeds of M.* pruriens *were procured (2010) locally after authentication, and a voucher specimen (herbarium voucher No. 6907) was deposited in the Department of Plant Biology and Plant Biotechnology (The Presidency College, Chennai, India). The seeds were washed using tap water and distilled water. The seeds were allowed to dry in the shade for two weeks. Then, the seeds were crushed into coarse powder and transferred into a container, and ethanol was added as a solvent until the coarse particles of the seed were completely soaked. The container was gently shaken every one-hour interval for 72 hr (until the solvent became colorless) and the filtrate was lyophilized (Harborne, 1973 ▶). The yield was around 20-22% per kg of seed and extract was subjected to high-performance liquid chromatography (HPCL) analyses. 

In our previous study, three different doses were tried, i.e. 150, 200 and 250 mg/kg. The 200 mg dose exhibited aphrodisiac, androgenic and spermatogenic properties without adverse side effects (Suresh et al., 2009 ▶), and our later studies also confirmed the effectiveness of this dosage (Suresh et al., 2013 ▶; Suresh and Prakash, 2011 ▶, 2012; Prakash et al., 2018 ▶). Hence, extract at a dose of 200 mg/kg body weight was administered by gavage, daily.

**Serum analysis**

The blood samples were collected from the retro-orbital plexus. The blood collected was centrifuged, and serum was separated and used for testosterone (T), follicular stimulating hormone (FSH), and luteinizing hormone (LH) determination by radio-immunoassay and biochemical evaluation of lipid profile (total cholesterol, high-density lipoprotein (HDL), low-density lipoprotein (LDL), very-low-density lipoprotein (VLDL) and triglycerides). 

**Euthanasia and tissue harvesting**

 After completing experimental periods, the animals were euthanised by cervical dislocation. The carcass was dissected, and organs were collected and processed for histological and biochemical studies. For the histological study, tissue specimens were processed for paraffin embedding. Sections were stained with Ehrlich alum hematoxylin and eosin. Biochemical analyses are given in detail, *vide infra*.

**Morphometric and stereological investigations**

The conventional stereological principles and standard morphometric procedures were used to obtain quantitative information; details of our procedure have been described elsewhere (Prakash et al., 2007 ▶). The germinal thickness, volume fraction, numerical density, and tubular diameter were measured in various groups.

**Biochemical analysis of oxidative stress and antioxidant**

Tissue lipid peroxidation (LPO) level was estimated by the method of Ohkawa et al. (1979) ▶. The thiobarbituric acid reactive substance was expressed in nmol of malondialdehyde (MDA) formed ⁄ min ⁄mg protein. Superoxide Dismutase (SOD) activity in the tissue homogenate was estimated using the method of Marklund and Marklund (1974) ▶. The activity of catalase (CAT) was estimated by the method of Sinha (1972) ▶. Reduced glutathione (GSH) in the tissue homogenates was estimated by Ellman's method (1959) ▶. The glutathione-S-transferase (GST) activity was assayed by the method of Habig et al. (1974) ▶. Estimation of the activity of glutathione peroxidase (GPx) (glutathione: hydrogen-peroxide oxidoreductase) in the tissues was done by the methods of Rotruck et al. (1973) ▶ and Beutler et al. (1963) ▶.

Total reactive oxygen species (ROS) level was measured in testicular tissue and sperm using 2'7'-dichlorofluorescein diacetate (DCFH-DA) (Suresh et al., 2011 ▶). The superoxide anion production was measured by the method of Podczasy and Wei (1988) ▶. Hydroxyl radicals were measured according to the method described by Puntarulo and Cederbaum (1998) ▶.

**Sperm analysis**

The total ROS and sperm analyses were done on sperm collected from caudal epididymis. The following sperm parameters were analyzed: the concentration of the sperm, the viability of the sperm, sperm motility, morphology of the sperm, cytoplasmic droplet containing sperm, chromatin integrity of the sperm- acridine orange assay (details are given by Suresh et al., 2009 ▶). In addition, using the hypo-osmotic swelling test (HOST), the functional integrity of the sperm membrane was examined. 

**Statistical analysis**

The data collected were statistically analyzed using SPSS for Windows (SPSS, Version 16.0 Inc., Chicago, IL). The data were subjected to One-Way ANOVA, and the significance was determined using a “Tukey’s *post hoc*” test with p<0.05 considered statistically significant. Data are presented as mean±SEM.

## Results

**Serum hormone and cholesterol analysis**

**Figure 1:**
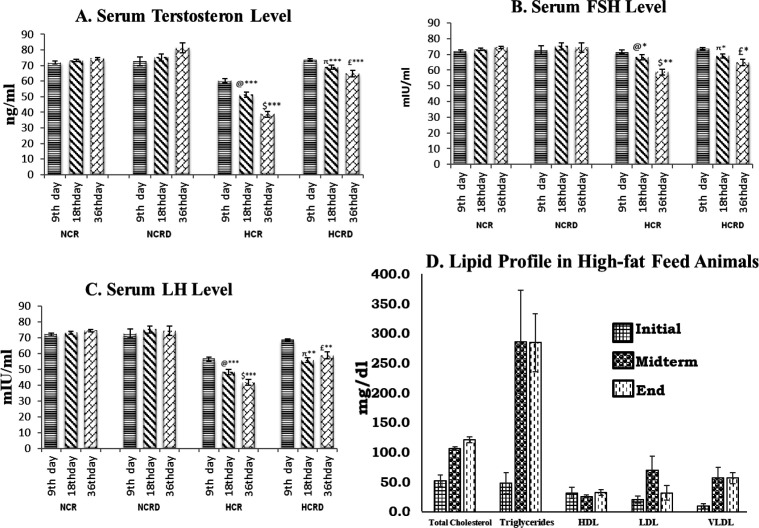
Illustrates serum hormone level. (A) Testosterone, (B) Follicular stimulating hormone and (C) Luteinizing hormone, in various experimental groups (n=6). @ - Control compared with other 18 days HCR group; $ - Control compared to 36 days HCR group; π – 18 days HCR compared to 18 days HCRD group; £ - 36 days HCR compared to 36 days HCRD group. *p<0.05; **p<0.01; and ***p<0.001. (D) Showing the lipid profile in high-fat diet-fed rats. Indicating the changes in various initial (0^th^ day), midterm (18^th^ day) and at the end of the experiment (36^th^ day). Each value represents mean±SEM (n=6). A significant increase in triglycerides, HDL, LDL, VLDL and total cholesterol levels were noted

Hormone levels were reduced in all HCR groups, T was reduced in 9 (not significant-NS), 18 (p<0.001), and 36 (p<0.001) days subgroups. The FSH was significantly reduced in 18 (p<0.05) and 36 (p<0.01) days subgroups. The LH level was significantly reduced in 18 (p<0.001), and 36 (p<0.001) days. In the case of high-fat fed animals treated with *M. pruriens* (HCRD group) showed a significant increase in T (p<0.001), FSH (p<0.05) and LH (p<0.01) levels in all the three subgroups (9, 18 and 36 days) (Figure 1A-C). The normal range of lipid profile in the initial phase (before the high-fat diet), was increased considerably after the high-fat diet intake (Figure 1D). A similar pattern was also seen in the HCRD rats.

**Histology and histometric analyses**

**Figure 2:**
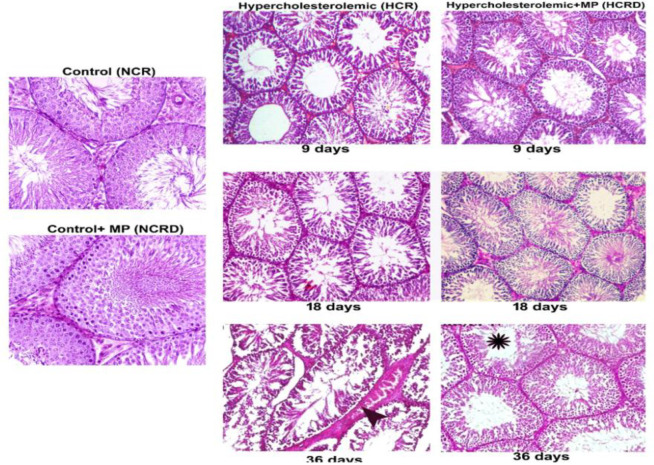
Photomicrograph of testis from various experimental groups. Control and control + *M. pruriens *(MP) rat testis demonstrating normal histoarchitecture. High-fat diet-fed rats indicate disturbance in spermatogenesis (arrowhead) and increased interstitium towards the 36^th^ day. These changes were reduced in hypercholesterolemic rats treated with *M. pruriens *(asterisk). (H & E, 40x)

**Figure 3:**
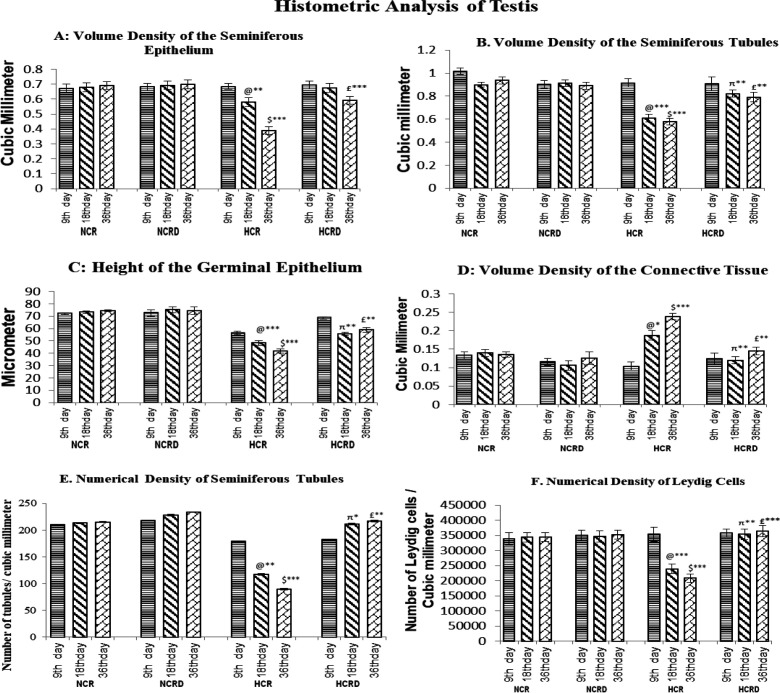
Illustrate testis histometric data collected from various experimental groups. A: Seminiferous diameter; B: Volume of the seminiferous epithelium; C: Height of germinal epithelium; D: Volume of connective tissue stroma; E: Number of seminiferous tubules per unit area; F: Number of Leydig cells per unit area. All values are in relative terms and expressed as mean±SEM (n=6). @ - Control compared with other 18 days HCR group; $ - Control compared to 36 days HCR group; π – 18 days HCR compared to 18 days HCRD group; £ - 36 days HCR compared to 36 days HCRD group. *p<0.05, **p<0.01 and ***p<0.001

Testis histology in control and HCR+*M. pruriens* groups normal histo-architecture. The testis harvested from 9 days high-fat diet-fed (HCR subgroup) rats showed detached germinal epithelial cells in the lumen. In 18- and 36-days subgroups of HCR rats, the testis histology showed tubular undulation, thickening of the basement membrane and interstitial degenerative changes and condensation of connective tissue around the blood vessel. These changes were considerably improved or reduced in the *M. pruriens*-treated group (Figure 2). Histometric data indicate significant disturbance in tubular diameter, tubular volume, epithelial and connective tissue components in all the three HCR subgroups but significant changes were seen in 18 days (p<0.001, NS, p<0.001 and p<0.05, respectively) and in 36 days (p<0.001, p<0.001; p<0.001 and p<0.001, respectively). The number of Leydig cells per mm^3^ was significantly decreased in HCR subgroups of 18 (p<0.01) and 36 days (p<0.01) (Figure 3)*. *All the above parameters showed improvement in the HCR+*M. pruriens* treated (HCRD) rats.

**The antioxidant level in testis**

**Figure 4:**
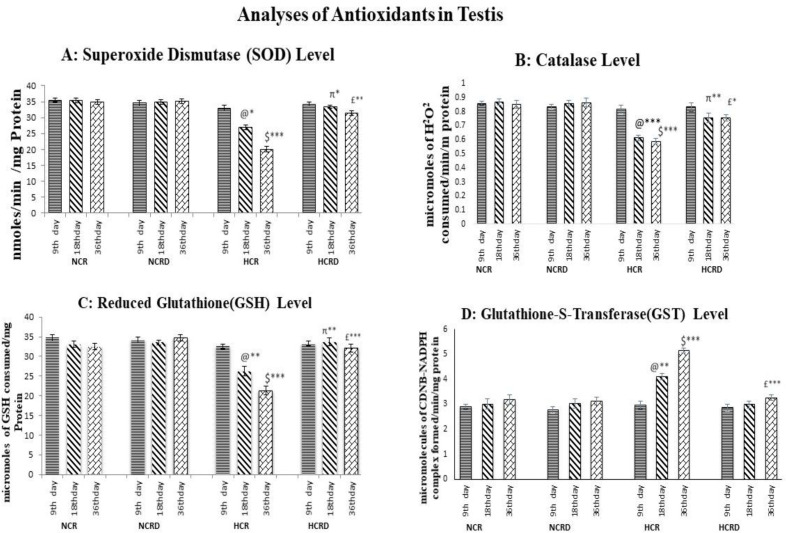
Illustrates the antioxidants level in the testis in various experimental groups. (A) superoxide dismutase (SOD), (B) Catalase activity, (C) Reduced Glutathione (GSH) activity, (D) Glutathione- S -Transferase level. Values are expressed as mean±SEM (n=6). @ - Control compared with other 18 days HCR group; $ - Control compared to 36 days HCR group; π – 18 days HCR compared to 18 days HCRD group; £ - 36 days HCR compared to 36 days HCRD group. *p<0.05, **p<0.01 and ***p<0.001

The SOD, CAT and GSH levels were reduced in all the three subgroups of HCR rats i.e., 9 (NS), 18 (p<0.05, p<0.001 and p<0.01, respectively), and 36 days (p<0.001, p<0.001 and p<0.001, respectively). These antioxidants were restored or elevated to the average in the HCR+*M. pruriens* group rats in 9 (NS), 18 (p<0.05, p<0.01 and p<0.01, respectively), and 36 days (p<0.01, p<0.05 and p<0.001, respectively). The GST activity was increased in all the HCR subgroups when compared with the control (Figure 4 A-D).

**Lipid peroxidation (LPO) and ROS level in testis**

**Figure 5:**
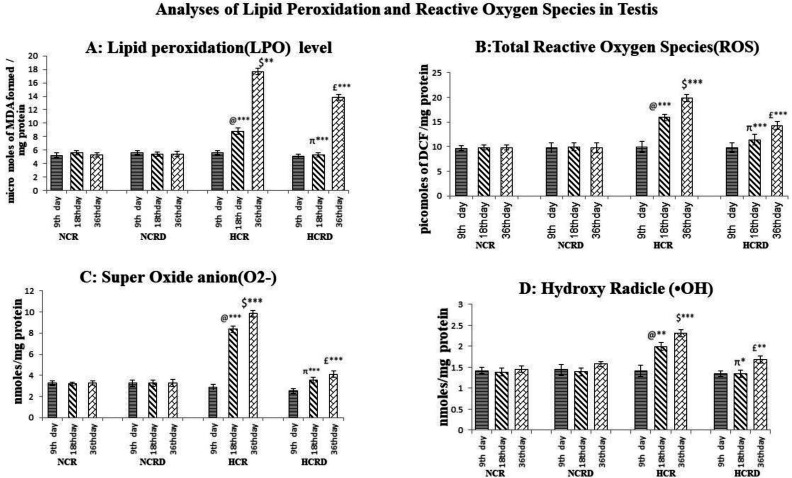
Shows the level of lipid peroxidation and reactive oxygen species in testis. (A) Lipid peroxidation level in the testis; (B) Total reactive oxygen species in testis (DCF assay); (C) Super oxide anion (O2-) in the testis; (D) Hydroxyl radical (•OH) in testis. Each value represents mean±SEM (n=6). @ - Control compared with other 18 days HCR group; $ - Control compared to 36 days HCR group; π – 18 days HCR compared to 18 days HCRD group; £ - 36 days HCR compared to 36 days HCRD group. *p<0.05, **p<0.01 and ***p<0.001

The MDA levels were increased in all the three subgroups of HCR i.e., 9 (NS), 18 (NS), and 36 (p<0.01) days compared to the control group. There is no significant change in total ROS level in the testis of the HCR, 9days subgroup but ROS level was increased significantly in 18 (p<0.01), and 36 days (p<0.001) of HCR subgroups compared to the corresponding control groups. Superoxide anion level was significantly increased in 18 (p<0.001), and 36 days (p<0.001) of HCR subgroups compared with corresponding control groups. Hydroxyl anion level was significantly increased in 18 (p<0.01), and 36 days (p<0.001) of HCR rat testis (Figure 5 A-D). The LPO and free radicals were significantly reduced in the HCR+*M. pruriens *group.

**Sperm analysis**

**Figure 6:**
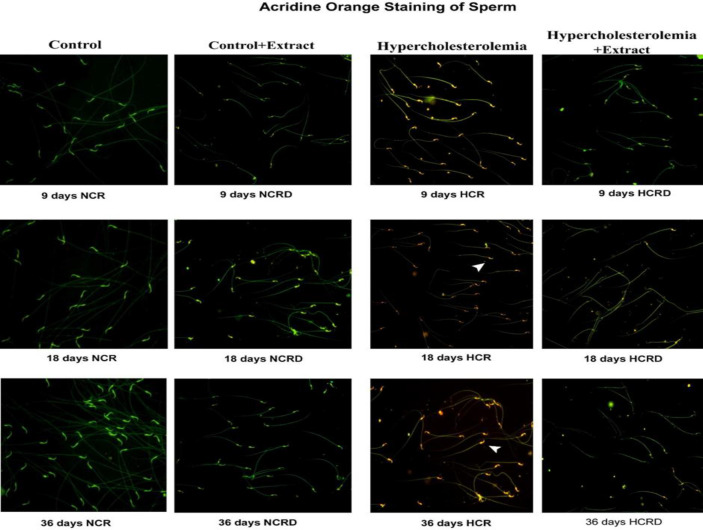
Photo-micrographic images of acridine orange (AO)-positive sperm cells in various experimental groups. AO staining of HCR rats sperm in orange-red colour (arrowhead) indicates significantly reduced chromatin integrity, especially in the long-term HCR group. This change was reduced in hypercholesterolemic rats treated with *M. pruriens.* (20X)

**Figure 7:**
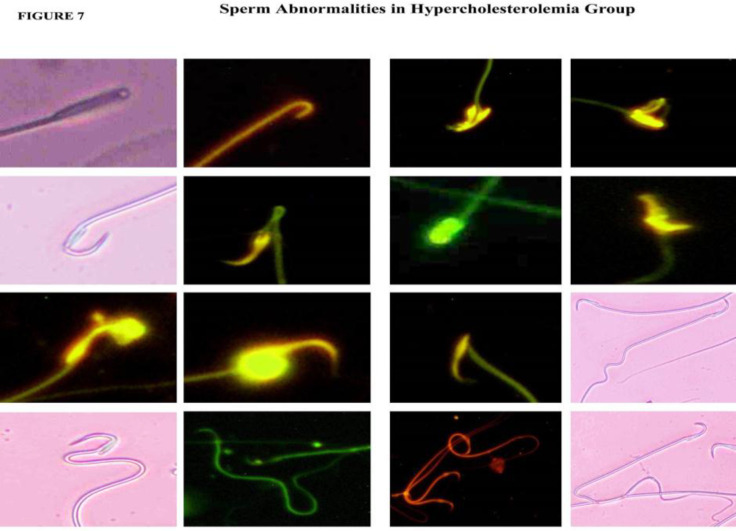
Light micrographic and fluorescent (AO) images of abnormal sperm morphology with head and tail defects from hypercholesterolemic rats

**Figure 8:**
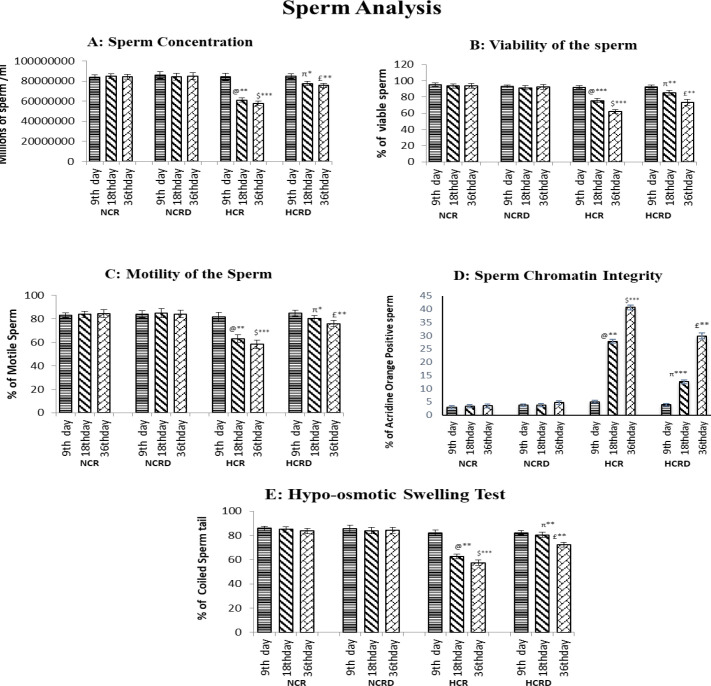
Illustrates various sperm parameters. (A) Concentration, (B) Viability, (C) Motility, (D) Chromatin integrity (Acridine orange assay), (E) Percentage of tail coiling in sperm cells (hypo-osmotic swelling test). Value represents mean±SEM (n=6). @ - Control compared with other 18 days HCR group; $ - Control compared to 36 days HCR group; π – 18 days HCR compared to 18 days HCRD group; £ - 36 days HCR compared to 36 days HCRD group. *p<0.05, **p<0.01 and ***p<0.001

The sperm parameters indicate severe pathological changes in the HCR group, with significantly reduced sperm concentration in 18 days (p<0.01), and 36 (p<0.001) HCR subgroups. The motility and viability were also significantly reduced in 18 (p<0.001), and 36 days (p<0.001) HCR subgroups. The percentage of defective sperm (head and tail defects) and dead sperm number significantly increased. Increased chromatin damage was seen in 18 (p<0.001), and 36 days (p<0.001) and poor membrane integrity in 18 (p<0.01), and 36 days (p<0.001) was observed in all the three subgroups of HCR animals (Figure: 6 and 7). Sperm carrying cytoplasmic droplets/remnants was increased considerably in 18 (22%) and 36 days (33%) of HCR subgroups*.* These changes were reduced in HCR+*M. pruriens* rats (Figure 8).

## Discussion

This study used a high-fat diet to induce increased levels of lipid or cholesterol in the circulation and create a rat model for hypercholesterolemia, mimicking nutritional dyslipidemia or hypercholesterolemia (Whitfield et al., 2015 ▶). The high-fat diet-fed rats showed a gradual increase in testicular pathology from the 9^th^ to the 36^th^ day and exhibited disorganized germinal epithelium with closely apposed Sertoli cells and interstitial inflammatory changes. However, degenerative changes in the interstitial region were reduced in the *M. pruriens* treated high-fat feed rats. 

There was a poorly defined Leydig cells margin, and small eccentric nucleus and cellular number were reduced in HCR rats. This could be due to increased ROS and LPO leading to Leydig cell damage and reduced steroidogenesis (Murugesan et al., 2005 ▶), and poor spermatogenesis. The administration of *M. pruriens *induced restoration of Leydig cell's appearance and concomitant increases in androgen secretion, and improved spermatogenesis. In addition, *M. pruriens *is known for its androgenic potential (Suresh et al., 2010 ▶; Seppan et al., 2018 ▶). Histometric data revealed that germinal epithelial height and volume were reduced in HCR rat testis and reduced tubular diameter compared to the control group. These observations indicate spermatogenic impairment in HCR rat testis. In addition, HCR testis showed an increased connective tissue proportion. Interestingly, the degenerative changes were reduced in high-fat feed rats treated with *M. pruriens *(HCRD).

There was a significant increase in LPO in HCR rats testis and a concomitant elevated ROS. The deleterious effects of oxidative stress are counteracted by endogenous antioxidant enzymes, mainly superoxide dismutase (SOD) and catalase (CAT). The HCR group rats testis showed significant down-regulation in the activity of SOD and CAT leading to an increased level of O^-^_2_ and H_2_O_2_. On the other hand, *M. pruriens*-treated HCR group showed good CAT activity indicating antioxidant properties. The extract's ability to activate CAT to an active form and increase the bioavailability of CAT through chelation of free ions (Tripathi and Upadhyay, 2002 ▶) and by removing excess amount O^-^_2_ and H_2_O_2_ might have played a key role (Shukla et al., 2007).

GSH is a crucial component in cell growth and differentiation; the concentration of GSH decreased in the HCR groups compared to the control group. Decreased tissue GSH activities might be due to the increased utilization by up-regulated oxidative radicals. However, supplementation of *M. pruriens* in the HCRD significantly enhanced the GSH level in testis, indicating its antioxidant capacity. 

Glutathione S-transferases (GST) is a family of multifunctional detoxifying enzymatic antioxidants that catalyzes the conjugation of GSH with a large number of compounds (Kora et al., 1996 ▶). This enzyme activity was significantly elevated in HCR rats testis due to increased utilization of GSH by GST (Abou and Fouad, 2000 ▶). The increase in GST and decreased GSH activities followed by thiol depletion are important to sustaining pathogenic roles for oxidative stress (Das and Vasudevan, 2006 ▶). The supplementation of *M. pruriens* brought this enzyme level back to near normal, effectively maintaining cellular oxidation-reduction homeostasis.

The restoration or recovery from the oxidative state in HCR testis indicates that flavonoids and phenolic compounds in the extract exhibit high antioxidant and free-radical scavenging activities (Suresh et al., 2009 ▶). The seeds contain L-DOPA (Siddhuraju et al., 1996 ▶) which found to possess a strong antioxidant capacity and free radical scavenging activity (Kanazawa and Sakakibara, 2000 ▶). These activities were able to improve the cellular antioxidant system to overcome oxidative injury. Suppression of ROS production and an increase in free-radicals scavenging before they interact with the plasma membrane, might be influenced by the hydrogen sharing ability of *M. pruriens *(Pathania et al., 2020 ▶). Thus, reducing membrane damage by impeding self‐propagating LPO reaction.

Analyses of epididymal spermatozoa indicate that hypercholesterolemia affected sperm maturation in the post-testicular phase. Increased ROS induced sperm damage and abnormal morphology (head defects, neck, and tail defects) (Suresh et al., 2010 ▶); these changes were evident in 18 and 36 days subgroups of the HCR group. Uncontrolled production of ROS leads to oxidative stress and damage to sperm membrane lipids, proteins, and chromatin content (loss of DNA integrity) in HCR rats (Saez et al., 2013 ▶), and ultimately, sperm cells lose their ability to fertilize (Suresh et al., 2010 ▶). The high-fat diet and pathological changes seem to increase with the duration of exposure. The *M. pruriens*-treated HCRD rats showed reduced ROS due to antioxidant activity, and restoration of spermatogenesis reduced sperm pathology.

The HCR rats sperm showed reduced motility which might be due to increased ROS-induced rapid loss of intracellular ATP leading to a decrease in axonemal protein phosphorylation and sperm motility (Suresh et al., 2010 ▶). In addition, an increased cytoplasmic droplet or residual containing sperm in the HCR group could fuel ROS generation and oxidative stress (Suresh, 2010 ▶). Sperm collected from 18- and 36-days HCR rats showed diminished plasma membrane integrity in HOST. 

The HCR group rats showed a significant increase in AO-positive sperm percentage and indicated DNA damage or chromatin integrity loss due to oxidative stress. Due to the strong antioxidant property and free-radical scavenging potential of *M. pruriens, *it protected the peroxidation of the sperm membrane and improved sperm viability in the HCR model of rats.

Plasma testosterone levels were significantly reduced in various periods of high cholesterol exposure, i.e., 9, 18, and 36 days, and the concomitant reduction in LH and FSH levels occured in these groups. Hypercholesterolemia induces the release of endogenous opioids from the hypothalamus, which along with corticosteroids, suppresses hypothalamic gonadotrophin-releasing hormone (GnRH) and leads to reduced secretion of LH and FSH, and a decrease in testosterone (Ramaswamy and Weinbauer, 2015 ▶). However, hormone levels were improved in HCRD rats supplemented with *M. pruriens*, indicating its potential to increase androgen biosynthesis (Shukla et al., 2009 ▶) by activating the pituitary-testicular axis (Misra et al., 2004 ▶). Thus the potential of* M. pruriens* to reduce oxidative stress and Leydig cell damage could have rectified the perturbed testosterone, FSH, and LH levels (Suresh et al., 2009 ▶).

The high-fat diet rat model mimicked nutritional dyslipidaemia or hypercholesterolemia, and served as a good experimental animal model. The benefit of the high-fat diet-induced animal model is that it comprises a combination of genetic and dietary effects on the animal (Pollin and Quartuccio, 2013 ▶; Suleiman et al., 2019 ▶). Besides, the model creation is rapid and cost-effective, with a strict diet regimen, and the model could be consistent throughout the study. This model shows considerable similarity to human hypercholesterolemia as a result of a high-fat diet (Stanworth and Jones, 2009 ▶; Hariri and Thibault, 2010 ▶). There are limitations to the high-fat diet animal model. There is always a problem of abnormal overweight and obese during the model induction affecting the suitability of the model for the given research and the outcome becomes dubious. However, with properly standardized diet intake, determining periodic hormone and lipid profiles would help to develop a better obese model, especially in the long-term study. Moreover, the use of these experimental animal models has served to delineate the pathobiology associated with hypercholesterolemia and also paved the way for the advent of potential drugs and natural products. Thus, the high-fat diet animal model plays a crucial role in the development of pharmaceutical drugs and novel dietary interventions. Hence, the advantages of this experimental model overshadow the limitations. The deleterious effect of hypercholesterolemia on testis and sperm is multifactorial and constant. Hence, using a positive control for studying the exclusive role of antioxidants or anti-inflammatory or lipid-lowering agents is not adequate to check the multimode potential of the *M. pruriens*.

The beneficial effects of *M. pruriens* in HCRD rats were multifaceted i.e. spermatogenic, androgenic or steroidogenic, antioxidant and free-radical scavenging potential, and reduced lipid peroxidation. These results are encouraging to perform similar experiments focusing on protein and molecular aspects, and the outcome was inspiring to do a parallel study on humans.

## Conflicts of interest

The authors have declared that there is no conflict of interest.
